# Variability of discharge medical therapy for secondary prevention among patients with myocardial infarction with non-obstructive coronary arteries (MINOCA) in the United States

**DOI:** 10.1371/journal.pone.0255462

**Published:** 2021-08-02

**Authors:** Nathaniel R. Smilowitz, Rachel Dubner, Anne S. Hellkamp, Robert J. Widmer, Harmony R. Reynolds

**Affiliations:** 1 Leon H. Charney Division of Cardiology, Department of Medicine, Soter Center for Women’s Cardiovascular Research, New York University School of Medicine, New York, NY, United States of America; 2 Duke Clinical Research Institute, Durham, NC, United States of America; 3 Baylor Scott & White Medical Center, Temple, TX, United States of America; University of Bologna, ITALY

## Abstract

**Background:**

Optimal medical therapy after myocardial infarction with nonobstructive coronary arteries (MINOCA; <50% stenosis) is uncertain. We evaluated variability in discharge prescription of angiotensin-converting enzyme inhibitors / angiotensin receptor blockers (ACEI/ARB) and beta-blockers (BB) to MINOCA patients between hospitals to assess physician equipoise about secondary prevention.

**Methods:**

Patients with MINOCA between 2007–2014 were identified in the NCDR Chest Pain–MI Registry. Those with prior revascularization or missing demographic, angiographic, or medication data were excluded. Analysis was limited to high-volume hospitals with ≥20 MINOCA total discharges. Discharge prescriptions for ACEI/ARB and BB after MINOCA were analyzed for each hospital. Clinical data on left ventricular ejection fraction (LVEF), glomerular filtration rate (GFR), and diabetes mellitus status were extracted to identify other indications for ACEI/ARB or BB.

**Results:**

Clinical data were available for 17,849 MINOCA patients, of whom 8,752 (49%) had LVEF <40%, GFR ≤60 mL/min, and/or diabetes. 5,913 patients without one of these indications for ACEI/ARB or BB were discharged from 156 high-volume hospitals. At discharge, ACEI/ARB was prescribed to between 16.0% and 88.8% of MINOCA patients (median 45.6%, IQR 38.0%-56.5%) and BB to between 28.0% and 97.5% (median 74.1%, IQR 64.7%-80.0%).

**Conclusion:**

There is marked variability between hospitals in the proportions of patients receiving ACEI/ARB and BB after hospitalization for MINOCA, suggesting clinical equipoise about the routine use of these agents. Randomized clinical trials are necessary to establish the benefit of ACEI/ARB and BB to improve outcomes after MINOCA.

## Background

Myocardial infarction (MI) with nonobstructive coronary arteries (MINOCA) accounts for approximately 6% of all MI [1]. Patients with MINOCA are more likely to be female, younger, and less likely to present with ST-segment-elevation MI (STEMI) compared to MI patients with obstructive coronary artery disease (MI-CAD) [2]. According to the ESC and AHA international, standardized definition of MINOCA, diagnosis requires: (1) fulfillment of MI criteria as outlined by the Fourth Universal Definition of MI [3], (2) nonobstructive coronary arteries, defined as <50% stenosis in all major epicardial vessels, and (3) no other clinically apparent cause for the acute presentation [1, 4]. The pathophysiology of MINOCA is heterogeneous and may differ from that of MI-CAD. MI-CAD is typically caused by unstable plaque; in contrast, mechanisms of MINOCA may include plaque rupture or erosion, coronary thrombosis or thromboembolism, coronary artery spasm, and, less commonly, spontaneous coronary artery dissection (SCAD). Although MINOCA is associated with a more favorable prognosis than MI-CAD, long-term outcomes are still poor [1, 4]. In the large SWEDEHEART registry, 13% of MINOCA patients died and 24% experienced a major adverse cardiac event (MACE) during a mean follow-up of 4 years [5].

Optimal medical therapy for secondary prevention of cardiovascular events after MINOCA is uncertain, because no randomized clinical trials have been conducted in this population. In particular, the role of angiotensin-conversing enzyme inhibitors (ACEI/)/angiotensin receptor blockers (ARB) and beta-blockers (BB) is not clear. These agents are typically prescribed with the goal of mitigating consequences of left ventricular dysfunction after MI, such as heart failure, adverse remodeling, and sudden cardiac death. These are less common after MINOCA than MI-CAD [6]. Observational data from the SWEDEHEART registry indicated that MINOCA patients who received ACEI or ARB at discharge had better long-term outcomes than those who did not. There was also a trend towards better outcomes among MINOCA patients who received BB at discharge [5]. The SWEDEHEART analysis did not show any difference in outcomes between propensity-matched groups of MINOCA patients who did and did not receive dual antiplatelet therapy; use of aspirin was nearly universal, so effects of aspirin could not be explored [5]. In the absence of data from clinical trials, clinical practice guidelines provide few recommendations for the management of patients with MINOCA, and discharge prescription rates for ACEI/ARB and BB are lower after MINOCA than after MI-CAD [5]. Some MINOCA patients have indications for one or both of these therapies, such as reduced left ventricular ejection fraction (LVEF), diabetes mellitus (DM) with proteinuria, or chronic kidney disease (CKD) [2]. However, many do not. In this study, we aimed to evaluate the variability in discharge prescription rates of ACEI/ARB and BB to MINOCA patients between hospitals to understand the extent to which there is equipoise in the medical community about the use of these agents after MINOCA. Understanding use patterns for ACEI/ARB and BB may provide insights into provider attitudes regarding whether clinical practice guidelines for MI apply to MINOCA patients.

## Methods

Data were obtained from the American College of Cardiology (ACC) National Cardiovascular Data Registry (NCDR) Chest Pain MI Registry (formerly, ACTION Registry–GWTG) [7]. The registry includes clinical data on MI patients from over 750 hospitals in the United States. A total of 596,820 adults who underwent coronary angiography for STEMI and non-ST-segment-elevation MI (NSTEMI) between January 1, 2007 and December 31, 2014 were identified in the registry. Patients included in The NCDR Chest Pain MI registry had ischemic symptoms at rest, lasting ≥10 minutes, and occurring within 24 hours before admission (or up to 72 hours for STEMI), and ECG changes consistent with STEMI or abnormal cardiac biomarkers associated with NSTEMI within 24 hours after the initial presentation [7]. Those with missing coronary angiographic data (n = 61,218), prior percutaneous coronary intervention or coronary artery bypass grafting (n = 186,829), thrombolytic therapy (n = 12,076), cocaine use (n = 3,632), cardiac arrest at presentation (n = 9,999), or missing age or gender data (n = 543) were excluded from the analysis. MINOCA was defined as <50% stenosis in all major epicardial vessels; patients with a stenosis ≥50% were excluded (n = 303,605). Patients missing discharge medication data were also excluded (n = 555). We have previously reported the incidence and in-hospital outcomes of MINOCA from the Chest Pain MI Registry using these methods [2]. The present analysis was limited to centers that discharged ≥20 MINOCA patients during the study period. The proportions of discharge prescriptions for ACEI/ARB and BB were obtained for each participating hospital. Clinical data on LVEF measured closest to discharge, estimated glomerular filtration rate (eGFR), CKD status (eGFR ≤60 mL/min), and DM status were extracted for all patients to identify those with existing indications for ACEI/ARB or BB. Center and variability of continuous variables were reported using mean, interquartile range (25^th^ and 75^th^ percentiles), and range (minimum and maximum values). Histograms were generated to illustrate the frequency distribution of the proportion of patients discharged on ACEI/ARB and BB by hospital. A waiver of written informed consent and authorization was granted by Chesapeake Research Review Incorporated. The Duke Clinical Research Institute served as the data-coordinating center and analyzed de-identified patient data.

## Results

There were 18,363 patients with MINOCA during the study period, among whom discharge therapy included aspirin in 85.3%, P2Y12 inhibitors in 27.2%, statins in 71.2%, ACEi/ARB in 57.9% and beta-blocker in 77.0% [2]. Among these patients, 15,615 were treated at one of 297 hospitals with at least 20 discharges with MINOCA during the study period. ACEI/ARB were prescribed to between 34.8% and 85.7% of MINOCA patients at discharge, with a median frequency of 58.3% (IQR 51.9% - 66.0%). BB were prescribed to 48.0% to 97.1% of MINOCA patients, with a median frequency of 77.6% (IQR 72.4% - 83.3%).

Data on LVEF, eGFR, and DM status were available for 17,849 MINOCA patients, among whom 8,754 (49%) had LVEF < 40%, CKD, and/or DM. Among patients who did not have one of these indications for ACEI/ARB and/or BB, 5,913 patients were discharged from 156 high-volume hospitals. These patients represent the final study cohort.

**Fig 1:**
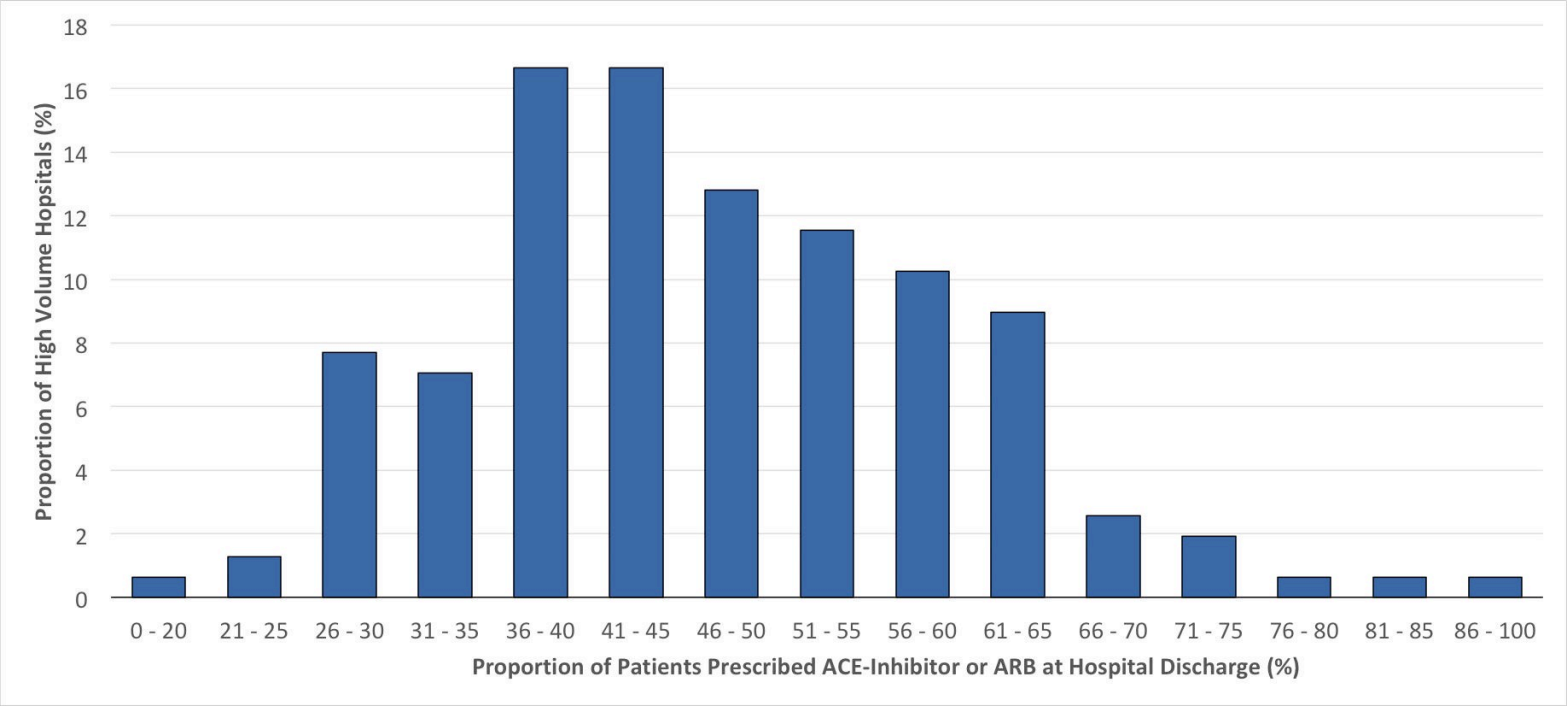
Frequency distribution of the proportions of patients without common indications for ACEI/ARB and/or BB (LVEF <40%, eGFR ≤60 mL/min, diabetes mellitus) who were prescribed ACEI/ARB at hospital discharge*. * Data from hospitals with ≥20 discharges with MINOCA during the study period.

**Fig 2:**
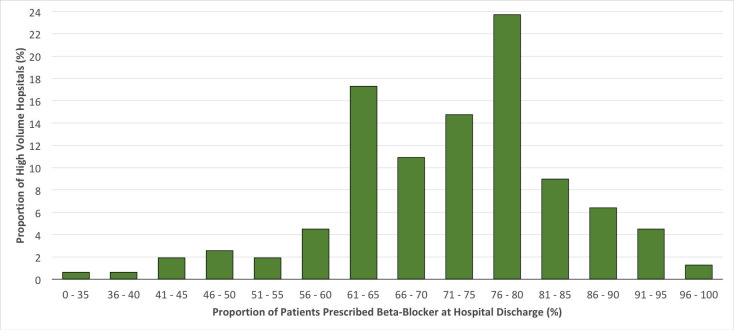
Frequency distribution of the proportions of patients without common indications for BB and/or ACEI/ARB (LVEF <40%, eGFR ≤60 mL/min, diabetes mellitus) who were prescribed BB at hospital discharge*. * Data from hospitals with ≥20 discharges with MINOCA during the study period.

At discharge, participating high-volume hospitals prescribed ACEI/ARB to anywhere between 16.0% to 88.8% of MINOCA patients without DM, CKD or reduced LVEF, with a median frequency of 45.6% (IQR 38.0% - 56.5%) (**Fig 1**). These same hospitals prescribed BB to anywhere between 28.0% and 97.5% of MINOCA patients, with a median of 74.1% (IQR of 64.7% to 80.0%) (**Fig 2**).

## Discussion

In this analysis of MINOCA patients included in the NCDR Chest Pain-MI Registry, we identified substantial variability between hospitals in the proportions of patients prescribed ACEI/ARB and BB. Prior studies indicate that MINOCA patients are less likely than MI-CAD patients to receive all secondary prevention medications upon discharge [2, 8]. Treatment variability by hospital was even more pronounced among patients without specific indications for ACEI/ARB and BB. These findings reflect a lack of consensus among physicians about the use of these post-MI secondary prevention in MINOCA patients, most likely related to the varied underlying mechanisms of MINOCA.

Observational data suggest ACEI or ARB may improve long-term outcomes in patients with MINOCA [5, 9]. There are plausible mechanisms for benefit, since ACEI/ARB improve endothelial function. Effects on endothelial function may be most beneficial when coronary artery spasm is a cause of or contributor to MINOCA pathogenesis, or when atherosclerotic disease is present. Prior observational studies also suggest beneficial effects of ACEI/ARB therapy in microvascular coronary artery disease and in takotsubo syndrome [10, 11].

Beta-blockers may provide benefit by reducing myocardial oxygen demand in patients with MI. Anti-ischemic effects of BB may or may not be relevant in MINOCA because the infarct artery is patent. Anti-arrhythmic properties of BB may also be beneficial in counteracting the downstream consequences of infarction, such as increased risk of ventricular arrhythmias and sudden cardiac death post-MI. Both ACEI/ARB and BB may reduce ventricular remodeling after MI and limit negative consequences of the infarction itself, irrespective of the initial mechanism of infarction. Still, in the reperfusion era, the benefit of BB treatment in patients with MI-CAD and preserved LVEF is controversial and the role for BB in patients with MINOCA is unknown [12]. In a retrospective multicenter Italian study of patients with MINOCA, BB use was associated with a reduction in acute coronary syndrome, heart failure hospitalization, stroke, or death [13].

In the absence of MI, approximately half of MINOCA patients in the Chest Pain MI Registry do not have a specific indication for ACEI/ARB or BB. This is important because of the potential risk of adverse effects associated with ACEI/ARB and BB. Adverse effects of ACEI/ARB may include persistent cough, angioedema, hyperkalemia, renal insufficiency and hypotension; adverse effects of BB include exacerbation of asthma, fatigue, and sexual dysfunction. Therefore, clinical trials are necessary to determine whether the benefits of medical therapy with ACEI/ARB and BB outweigh risks of adverse effects in MINOCA patients. Data from this analysis provides additional justification for the Randomized Evaluation of BB and ACE/ARB Treatment in MINOCA Patients (MINOCA-BAT) clinical trial, which is currently ongoing in Sweden and Australia to evaluate the clinical benefit of these agents in MINOCA [14]. In contrast to BB and ACE/ARB, physicians may have a lower threshold to prescribe aspirin and statin therapy for MINOCA because these agents reduce short and long term cardiovascular risk in other populations, including patients with MI-CAD [15, 16]. Even so, patients with MINOCA in the United States are less likely to receive aspirin (85% vs. 97%) and statin (71% vs. 93%) at hospital discharge than patients with MI-CAD, highlighting equipoise over the role for aspirin and statin therapy in MINOCA and opportunities to improve quality of care for these patients.

There are some limitations of this analysis. The NCDR Chest Pain MI registry only includes patients referred for coronary angiography at centers in the United States, and prescribing patterns may vary internationally. Since all participating hospitals enroll in the registry voluntarily, the study cohort may not be representative of all hospitals. We were unable to review medical records to verify presumed MINOCA diagnoses; however, the directions for inclusion in the NCDR Chest Pain MI registry require a clinical diagnosis of MI, and the proportion of MI patients with MINOCA in this cohort is similar to multiple previously published large studies [2, 17] Additionally, results of diagnostic testing performed after coronary angiography were not captured in the registry; a minority of patients with MINOCA may be found to have myocarditis or another diagnosis on cardiac MRI [18]. However, typical practice at sites is to enter cases into the registry at or after the time of discharge, when the final diagnosis has been established [7]. Cardiac MRI was not part of guideline recommendations for MINOCA during the study period. Lastly, although common indications for ACEI/ARB and BB were taken into account in (LVEF <40%, CKD, DM), other possible indications (e.g. arrhythmia) and contraindications (e.g. drug allergies) were not recorded in the registry.

In conclusion, marked variability between hospitals in the proportions of patients receiving ACEI/ARB and BB after hospitalization for MINOCA suggests clinical equipoise about the routine use of these agents. Randomized clinical trials are necessary to establish the benefit of ACEI/ARB and BB to improve outcomes after MINOCA.
